# Impact and economic analysis of human T-cell lymphotropic virus type 1 (HTLV-1)-targeted antenatal screening, England and Wales, 2021

**DOI:** 10.2807/1560-7917.ES.2024.29.22.2300537

**Published:** 2024-05-30

**Authors:** Carolina Rosadas, Milene Costa, Kátia Senna, Marisa Santos, Graham P Taylor

**Affiliations:** 1 Section of Virology, Department of Infectious Disease, Faculty of Medicine, Imperial College London, London, United Kingdom; 2 Núcleo de Avaliação de Tecnologias em Saúde, Instituto Nacional de Cardiologia, Rio de Janeiro, Brazil; 3 Faculdade de Farmácia, Universidade Federal do Rio de Janeiro, Rio de Janeiro, Brazil; 4 National Centre for Human Retrovirology, Imperial College Healthcare NHS Trust, London, United Kingdom

**Keywords:** HTLV, antenatal screening, economic analysis, cost-utility analysis, vertical transmission, prevention, mother-to-child transmission, maternal health

## Abstract

**Background:**

Human T-cell lymphotropic virus type 1 (HTLV-1) is a neglected virus that can cause severe disease and be transmitted from mother to child through breastfeeding. Avoidance of breastfeeding prevents 80% of vertical transmission. The United Kingdom (UK) is currently assessing whether HTLV-1-targeted antenatal screening should be implemented.

**Aim:**

We aimed to assess the impact and cost-effectiveness of a targeted programme to prevent HTLV-1 vertical transmission in England and Wales.

**Methods:**

We estimated the number of pregnant women who have high risk of HTLV-1 infection based on their or their partner’s country of birth. With data from 2021, we used a mathematical model to assess cost-effectiveness of HTLV-1 antenatal screening. We also estimated the annual number of infant infections and the number that could be prevented with screening and intervention.

**Results:**

We estimate that ca 99,000 pregnant women in England and Wales have high risk of HTLV-1 infection. In the absence of screening, 74 (range: 25–211) HTLV-1 infections in infants would be expected to occur every year in England and Wales. Implementation of targeted screening would prevent 58 (range: 19–164) infant infections annually. The intervention is effective (incremental 0.00333 quality-adjusted life years (QALY)) and cost-saving (GBP −57.56 (EUR −66.85)).

**Conclusion:**

Our findings support implementation of HTLV-1 targeted antenatal screening to reduce vertical transmission from mothers to infants in the UK.

**Key public health message**

**What did you want to address in this study and why?**

HTLV-1 is a neglected virus that can cause severe disease, including an aggressive leukaemia and disabling neurological conditions. Virus transmission from mother to child can occur through breastfeeding, but exclusive formula feeding can prevent 80% of infections. Few countries have implemented policies for HTLV-1 screening. We wanted to assess the impact and cost-effectiveness of targeted antenatal HTLV-1 screening in the United Kingdom (UK).

**What have we learnt from this study?**

We learnt that each year around 99,000 pregnant women in England and Wales are at high risk of living with HTLV-1 because of their or their partner’s origin from high endemic areas. A targeted antenatal screening approach would prevent between 19 and 164 infant infections every year. The economic analysis not only confirmed the proposed policy would be cost-effective but showed that such an approach would be cost saving.

**What are the implications of your findings for public health?**

Our analysis provides support for implementation of interventions to prevent HTLV-1 mother-to-child transmission in the UK and other European countries with similar scenarios, i.e. low endemic countries with high numbers of immigrants from high prevalence countries. Our findings can also help to support the emerging global response to HTLV-1 infection initiated by the World Health Organization and improve health equity.

## Introduction

Antenatal and newborn screening for various infectious and non-infectious diseases are in place to ensure both maternal and child optimal health. In the United Kingdom (UK), pregnant women are offered screening for infectious diseases (hepatitis B, HIV and syphilis), inherited diseases (sickle cell, thalassaemia and other haemoglobin disorders), genetic conditions (Down's, Edward’s and Patau's syndromes) and 11 physical conditions [1]. Additionally, newborns are routinely screened for nine rare but serious conditions (listed in Supplementary Table S1) [1]. The UK National Screening Committee advises on which antenatal and newborn screening policies should be implemented in the country [2]. However, given the large number of screening tests that are currently available, the decision as to which tests to include in the context of limited resources can be challenging.

HTLV-1 is a neglected retrovirus that causes a chronic lifelong infection that can lead to many diseases, including severe leukaemia and progressive and disabling neuroinflammation (HTLV-1-associated myelopathy, HAM). People living with HTLV-1 have a 57% increase in the risk of dying by age group compared with the general population [3]. HTLV-1 is transmitted by condomless sex and contact with infected blood cells and organs, but also passed vertically from mother-to-child mainly through breastfeeding. About 4% of all those infected with HTLV-1 will develop adult T-cell Leukaemia/Lymphoma (ATL), the most severe disease caused by HTLV-1, with a median survival of less than 1 year [4]. The risk of developing ATL is estimated to be as high as 20% following early life infection. Recently, the World Health Organization (WHO) has recognised human T lymphotropic virus type 1 (HTLV-1) as a global concern and prevention of mother-to-child transmission is one of the goals for 2030. [5,6].

Global prevalence of HTLV-1 differs widely between and within countries. In some countries in South America and the Caribbean, HTLV-1 prevalence reaches 4% in pregnant women, but is higher in women aged over 40 years, with up to 25% of this group living with HTLV-1 in rural areas of Africa [7]. In most European countries where HTLV-1 prevalence is known, overall prevalence is considered low, according to the threshold proposed by European Centre for Disease Prevention and Control (ECDC) (below 1/10,000 among first-time blood donors and/or below 1% in the general adult population) [8]. However, in Romania, HTLV-1 prevalence in first-time blood donors is 5.3 per 10,000 [9], and in Moldova, 95 per 10,000 blood donors are infected [10]. In the UK and Spain, most people living with HTLV-1 are from ethnic minority groups originating from high prevalence countries [11]. Overall HTLV-1 prevalence in pregnant women in the UK is 0.053%, while prevalence in mothers born in the Caribbean reaches 1.7% [12]. 

Most people living with HTLV-1 worldwide, including those of reproductive age, are unaware of their infection and lack of public awareness and screening contributes to this situation. HTLV-1-associated diseases usually arise during adulthood, i.e. 4–5^th^ decades of life. Symptoms are often misdiagnosed and ascribed to other conditions. There is no vaccine nor curative treatment for this persistent infection. Prevention of transmission still relies on safer sex and screening of blood and organ donors [6]. 

Preventive strategies to avoid vertical transmission are based on shortening or avoidance of breastfeeding. Exclusive formula feeding prevents around 80% of infant HTLV-1 infections and is recommended in many countries [13]. However, national HTLV-1 antenatal screening is still limited to Japan [6] and Saint Lucia [14]. Although the UK National Screening Committee has previously advised against the implementation of universal HTLV-1 antenatal screening in the country, targeted HTLV-1 testing for pregnant women who are at high risk of living with this virus is currently being assessed [15]. The primary aim of our study is thus to evaluate whether targeted HTLV-1 antenatal screening is cost effective in the UK. Secondary aims are to calculate (i) the number of infants that would be infected every year in the absence of antenatal screening and (ii) the annual number of infant infections that HTLV-1 antenatal targeted screening followed by intervention would prevent.

## Methods

### Estimation of number of pregnant women at high risk of HTLV-1 infection

#### Data extraction

For all live births in England and Wales during 2021, data on country of birth of each parent were exported from government statistics [16]. Information on parent country of birth for live births was not available for Northern Ireland and Scotland. Therefore, our analysis has focused on England and Wales.

For parents born outside the UK, data were aggregated by the following regions/countries: European Union (EU), rest of Europe (non-EU), Africa (North, Western, Central and Southern Africa), Americas and the Caribbean (North America, Central America, South America and Caribbean), Middle East and Asia (Middle East, Central Asia, Eastern Asia, Southern Asia (India, Pakistan, Bangladesh) and Southeastern Asia), Antarctica and Oceania (Australasia and Other Oceania) [16]. 

The number of live births for the 10 most common countries of birth for non-UK-born pregnant women in England and Wales in 2021 was also extracted and is presented in Supplementary Table S3 [16]. 

#### Classification of high/low HTLV-1 prevalence

Countries/regions were classified dichotomously as areas with high or low HTLV-1 prevalence according to the ECDC definitions and report [8]. In addition, recent data from WHO on the prevalence of HTLV-1 among blood donors was used to assess whether any regions/countries would now be classified as high prevalence based on data not available in the ECDC report [17]. HTLV-1 prevalence data are unknown for some countries/areas, therefore, a consensus among authors was used, when needed.

The number of pregnant women considered having high risk of being infected by HTLV-1 was calculated based on the woman’s country of birth or for those women who were not born in high prevalence areas, on the country of birth of the paternal parent. The total number of infants born from parents originating from high prevalence areas were aggregated in an excel spreadsheet.

### Estimation of HTLV-1 infant infections prevented by antenatal screening

The estimated number of women at high risk of HTLV-1 infection in England and Wales was inputted in the cost-utility model developed by our group [18] to calculate the number of infants that would be infected every year in the absence of antenatal screening and the annual number of infant infections that HTLV-1 antenatal targeted screening followed by exclusive formula feeding would prevent. 

This estimate of HTLV-1 infant infections considers the following six variables: (i) attributable risk of mother-to-child HTLV-1 transmission (residual vertical transmission in the absence of breastfeeding, i.e. transmission rate during pregnancy or delivery, transmission rates if infants are breastfed for up to 6 months and if breastfeeding occurs for more than 6 months); (ii) prevalence of HTLV-1 in pregnant women born in high endemic areas; (iii) incidence of breastfeeding in the UK (black women) [19]; (iv) prevalence of breastfeeding at 6 months (black women in the UK) [19]; (v) number of women with high risk of HTLV-1 infection; and (vi) performance of diagnostic tests (sensitivity and specificity). National data about infant feeding in the UK is stratified by ethnicity (White, Asian, Black, Chinese or other ethnic origin). We opted to use data related to Black women as this is more representative of the UK cohort.

### Cost-utility analysis

A cost-utility analysis was performed to compare non-intervention, i.e. without HTLV-1 antenatal screening, to the intervention scenario: inclusion of targeted HTLV-1 antenatal screening for women at high HTLV-1 risk followed by intervention, which consisted of cabergoline, a drug used to prevent the initiation of lactation (one single dose of 1 g tablet by mouth), and provision of infant formula for 6 months for those living with the virus [19].

**Figure 1:**
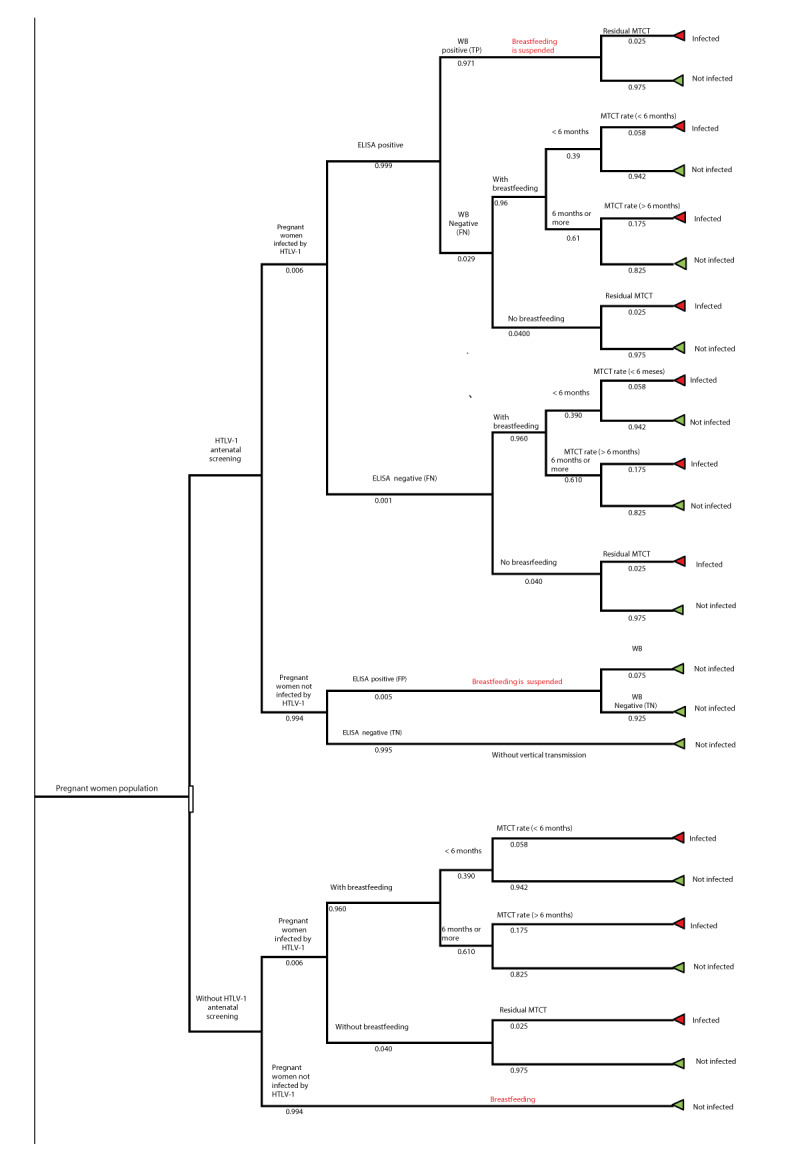
Decision tree used in the hybrid model to determine the cost-effectiveness of targeted HTLV-1 antenatal screening in England and Wales

ELISA: enzyme linked immunoassay; FN: false negative; FP: false positive; HTLV-1: human T-cell lymphotropic virus type 1; MTCT: mother-to-child transmission; TN: true negative; TP: true positive; WB: Western blot.

**Figure 2:**
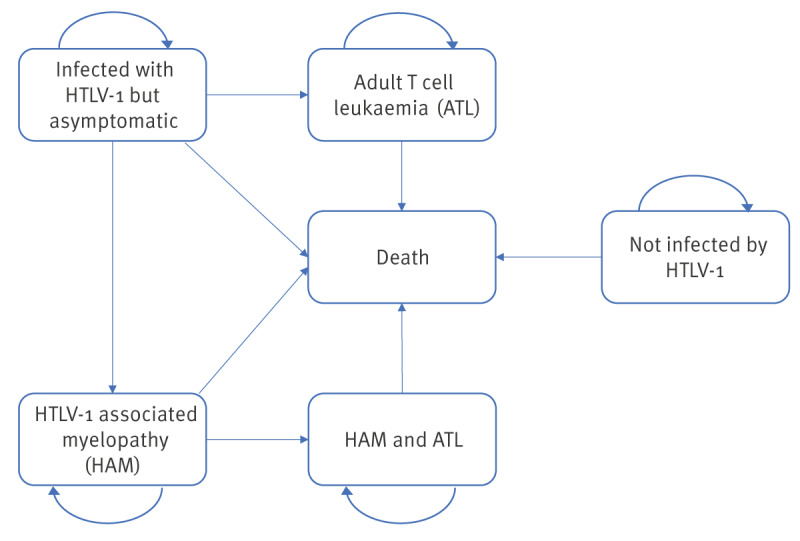
Markov model showing possible outcomes incorporated in the cost-effectiveness analysis of targeted HTLV-1 antenatal screening in England and Wales

ATL: adult T-cell leukaemia; HAM: HTLV-1-associated myelopathy; HTLV-1: Human T-cell lymphotropic virus type 1.

Cost-utility analysis was conducted using the editable tool that was previously developed by our group [18]. This tool comprises a decision tree (Figure 1) combined with Markov model (Figure 2). The decision tree captures the number of mother-to-child infections that happen in the two different scenarios (non-intervention vs intervention) being assessed. The Markov model captures the lifetime outcomes possible for an infant: (i) not infected by HTLV-1, (ii) infected with HTLV-1 but asymptomatic, (iii) patients with HAM, (iv) patients with ATL, (v) patients with HAM and ATL and (iv) death. The perspective of the National Health Service (NHS) as a healthcare payer was used, and the time horizon was the infant’s lifetime.

This figure was adapted from Rosadas et al. [18].

### Model parameters

Data specific to the UK were obtained by literature review, by accessing government statistics or specialist advice. Model inputs are described in the Table. A detailed description of each input is provided in the Supplement. We have modelled with a discount at a fixed annual rate of 3% per year for costs and health effects, according to the National Institute for Health and Care Excellence (NICE)’s recommendation [20]. As an alternative, we have also modelled without a discount rate. All other parameters used in the model such as probability of HTLV-1 transmission according to duration of breastfeeding, performance of diagnostic tests and probability of developing HTLV-1 associated diseases were the same as used in the previous work [18], as they are not likely to be affected by local differences or are similar between the UK cohort (mainly Afro-Caribbean) and a Brazilian cohort [18].

**Table:** Parameters used in the model to assess the cost-effectiveness of target HTLV-1 antenatal screening in England and Wales

Parameter	Point estimate	Lower limit	Upper limit	Reference
Risk factors for transmission				
HTLV-1 prevalence in pregnant women from high prevalence areas	0.006	0.004	0.008	[12]
Prevalence of breastfeeding	0.960	0.864	1.000	[19]
Probability of breastfeeding for 6 months or more	0.610	0.549	0.671	[19]
Performance of diagnostic tests				
Sensitivity of ELISA	0.999	0.978	0.999	[18]
Specificity of ELISA	0.995	0.991	0.999	[18]
Sensitivity of Western blot	0.971	0.923	0.999	[18]
Specificity of Western blot	0.925	0.860	0.975	[18]
Probability of HTLV-1 transmission				
Probability of HTLV-1 mother-to-child transmission if breastfeeding lasts for < 6 months	0.058	0.046	0.070	[18]
Probability of HTLV-1 mother-to-child transmission if breastfeeding lasts for ≥ 6 months	0.175	0.140	0.210	[18]
Probability of residual HTLV-1 mother-to-child transmission without breastfeeding	0.025	0.020	0.050	[18]
Costs in GBP (EUR)				
Cost of ELISA (individually)	GPB 1.40 (EUR 1.63)	GBP 1.26 (EUR 1.46)	GBP 1.54 (EUR 1.79)	Product quote
Cost of Western blot (individually)	GBP 19.75 (EUR 22.94)	GBP 17.77 (EUR 20.64)	GBP 21.72 (EUR 25.22)	Product quote
Cost of breastfeeding interruption (cabergoline and formula) (per individual)	GBP 730.95 (EUR 848.86)	GBP 657.85 (EUR 763.97)	GBP 804.04 (EUR 933.74)	Estimated according to Methods
Cost of asymptomatic HTLV-1 infection (per individual/per year)	GBP 2,900 (EUR 3,367.80)	GBP 2,610 (EUR 3,031.02)	GBP 3,190 (EUR 3704.59)	Imperial College Healthcare NHS Trust Resource Group
Cost of HAM (per individual/per year)	GBP 16,000 (EUR 18,580.99)	GBP 14,400 (EUR 16,722.89)	GBP 17,600 (EUR 20,439.09)	Imperial College Healthcare NHS Trust Resource Group
Cost of ATL (per individual/year)	GBP 16,739.00 (EUR 19,439.20)	GBP 15,065.10 (EUR 17,490.64)	GBP 18,412.90 (EUR 21,383.12)	Imperial College Healthcare NHS Trust Resource Group
Risk of disease				
Relative risk of death of people living with HTLV-1 vs those without	1.570	1.370	1.800	[18]
Hazard ratio of death for people with HAM vs those without	5.030	1.959	12.911	[18]
Probability of death in people with ATL	0.2943	0.2713	0.3183	[18]
Probability of developing HAM	0.0053	0.0026	0.0109	[18]
Probability of developing ATL	0.0010	0.0006	0.0015	[18]
Probability of progression to ATL for people living with HAM	0.0038	0.0030	0.0046	[18]
Utility value				
Utility value of asymptomatic infection	0.780	0.702	0.858	[36]
Utility value of ATL	0.262	0.182	0.288	[18]
Utility value of HAM	0.203	0.182	0.223	[36]
Utility value without HTLV-1 infection	0.910	0.819	1.000	[36]
Discount rate	0.03	NA	NA	[20]

ATL: adult T-cell leukaemia; ELISA: enzyme-linked immunoassay; HAM: HTLV-1-associated myelopathy; HTLV-1: human T-cell lymphotropic virus type 1; NA: not applicable.

ELISA was used as an initial screening and Western blot as confirmatory test for HTLV-1 infection. All costs are reported in pound sterling (GBP) and Euros (GBP 1 = EUR 1.16, conversion date: 9 Apr 2024).

### Model assumptions

We made the following assumptions: confirmatory testing was by Western blot with no indeterminate results; onset of HTLV-1-associated diseases occurs from age 18 years or older; costs and outcomes for concurrent HAM and ATL state are the same as the ATL; ATL remission and maintenance treatment were not considered. Other HTLV-1-associated diseases other than HAM, ATL and the increase in overall mortality were not considered in the model because they are too complex to model and because of the uncertainty in the corresponding data.

### Measure of effectiveness

Quality-adjusted life-years (QALYs) was the measure of effectiveness used. QALYs express life expectancy adjusted for the quality of life during those years. The incremental cost-effectiveness ratio (ICER) was calculated per QALY gained by comparing proposed strategies and current practice and was the primary outcome of this study. The willingness-to-pay threshold was set as GBP 20,000 per QALY (EUR 23,227.30), as per the NICE`s guidance [20].

### Sensitivity analysis

To identify the impact of the uncertainties of each parameter in the obtained result, a one-way deterministic sensitivity analysis was performed. A probabilistic sensitivity analysis with 1,000 simulations was included to test the robustness of results. We assumed a beta probability distribution for prevalence, sensitivity and specificity of tests and probabilities, whereas we applied a log-normal distribution to relative risks and hazard ratios and a gamma distribution to costs.

## Results

### Number of pregnant women to be screened for HTLV-1

In our analysis, we determined the following regions/countries to be high HTLV-1 prevalence areas: Western Africa, Central Africa, Southern Africa, Central America, South America, Caribbean, Middle East, India, Pakistan and Romania. The HTLV-1 prevalence status in Southeast Asia is unknown, so pregnant women and partners born in this area were not included. Japan, Moldova and First Nations people from Australia have high prevalence of HTLV-1 infection, but pregnant women and partners in the UK born in these regions were not included as this information is not publicly available for the UK population. Similarly, women born in a low prevalence area whose partner was from Romania were not included, as we had no access to this information for the UK. Expectant mothers or partners that are first generation born in the UK from a family that has migrated from a high prevalence area should also be screened but were not considered in this analysis as this information was not available.

We calculated at least 98,996 of 624,828 pregnant women per year in England and Wales should be considered as having high risk of HTLV-1 infection and therefore, should be offered screening every year. A detailed description of the targeted population can be seen in Supplementary Table S2.

### Impact of targeted screening

Based on a mean HTLV-1 prevalence of 0.6% (range: 0.2–1.7) [12], the current pattern of breastfeeding in the UK and the performance of HTLV-1 diagnostics tests, we estimate that 74 (range: 25–211) infants born from mothers considered high risk are expected to be infected with HTLV-1 through breastfeeding every year in England and Wales. Targeted HTLV-1 antenatal screening, followed by intervention (exclusive formula feeding) could prevent 58 (range: 19–164) infant infections annually.

### Cost-utility of HTLV-1 targeted antenatal screening

**Figure 3:**
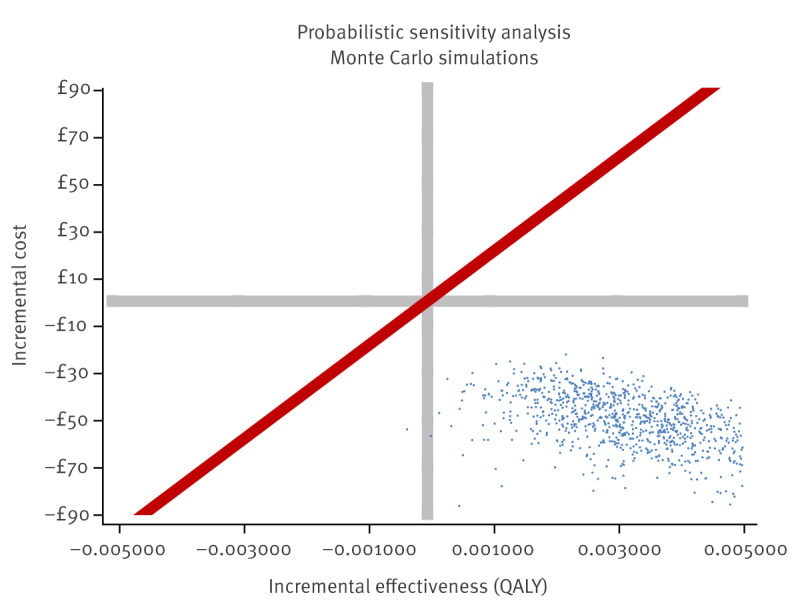
Probabilistic sensitivity analysis of the cost-effectiveness of targeted HTLV-1 antenatal screening in England and Wales

HTLV-1 targeted antenatal screening would produce a mean incremental health system cost of GBP −57.56 (EUR −66.85) per pregnant woman tested, indicating that the proposed intervention is cost-saving compared to current practice without screening. The incremental effectiveness was 0.00333 QALYs. Therefore, this would be deemed a dominant intervention, i.e. increases effectiveness and reduces cost. Sensitivity analysis showed that 99.9% of the simulations were dominant (Figure 3). If no discount rate is applied to costs and benefits, the incremental effectiveness is 0.0122 and the incremental costs GBP −159.06 (EUR −184.80), and all simulations were dominant in the sensitivity analysis.

QALY: Quality adjusted life years.

Red line represents the cost-effectiveness threshold, as recommended by NICE. Each blue dot represents the result of a Monte Carlo simulation. Costs are represented in pound sterling (GBP, £).

## Discussion

HTLV-1 can cause several severe diseases and can be transmitted from mother-to-child, predominantly through breastfeeding. Blood tests can identify expectant mothers living with HTLV-1, and guidelines are currently in place in the UK and many other countries to screen blood [10] and milk donations [13]. Interventions to prevent HTLV-1 mother-to-child transmission are still limited, but it is possible to prevent around 80% of infections if breastfeeding is replaced by infant formula. Although the UK National Screening Committee advised against universal HTLV-1 antenatal screening in 2022, targeted HTLV-1 antenatal screening, proposed in 2023, is now under consideration. [15]. The UK migrant`s health guidance already recommends testing people from high HTLV-1 endemic areas for HTLV-1 including pregnant or breastfeeding women [21], but this is not seen in clinical practice. In the present study, we have estimated that from 19 up to 164 new HTLV-1 infant infections could be prevented every year in England and Wales if targeted antenatal screening and intervention are implemented. 

Prevention of mother-to-child transmission will consequently avoid HTLV-1-associated diseases during childhood and adulthood and will provide an opportunity to signpost seropositive mothers and exposed babies to care. It will also potentially alleviate the burden that many mothers report feeling for having transmitted the virus to their babies, as they were unaware of their own infection by the time of birth. This has been consistently reported by patients, including patients from the UK [22]. Antenatal screening would also allow early diagnosis of pregnant women. With linkage to care, HTLV-1-associated diseases can be detected early enabling early treatment, which has been associated with improved disease outcome [23]. Counselling also provides opportunities to prevent ongoing transmission, not only through the vertical route but also horizontally, for example, by unprotected sex. The NHS in England already has a specialised, centrally commissioned clinical service providing care for people living with HTLV-1, The National Centre for Human Retrovirology, which could provide counselling and care for seropositive mothers and their babies [11].

A national HTLV antenatal screening programme has been implemented in Japan, a high prevalence country since 2010, following a successful programme in the region of Nagasaki [24]. A study conducted in 2023 confirmed that this policy is cost-effective in Japan [25]. Our group also demonstrated that universal screening is cost-effective in Brazil, a country that has antenatal screening implemented in some areas and has recently decided to implement national universal antenatal screening [18]. Previously we have shown that universal HTLV-1 screening could be cost-effective in the UK, if samples were assessed in pools [26]. In our study, we have demonstrated that targeted testing, using single samples, is cost-saving and more effective than not testing, in the UK. The finding of this study will also be of particular interest to other countries with similar scenarios, e.g. Spain, Sweden, the Netherlands and France – high-income countries with overall low prevalence of HTLV-1 infection in the general population, but with high prevalence in specific population groups. Thus, our results would be applicable if the costs of intervention and the impact of HTLV-1-associated diseases are similar and within the ranges used here [27]. The WHO reports that France is the only European country where HTLV-1 selective antenatal screening is offered but is restricted to French Caribbean territories [6]. HTLV-1 prevalence is usually higher in people living in vulnerable situations [28,29].

Regarding the prevention strategy, we have considered exclusive formula feeding in our model, as this is the most common recommendation for pregnant women living with HTLV-1. Recommendations to replace breastfeeding with infant formula should always consider whether the policy is affordable, feasible, acceptable, sustainable and safe (AFASS). There are no data on acceptance of this intervention among pregnant women in the UK. However, acceptance is reported to be high (> 90%) among people living with HTLV-1 in the country and in Japan [30,31], and this is what we observe in our clinical routine. Some argue that breastfeeding for up to 3 months may be an alternative to exclusive formula-feeding to reduce the risk of transmission, but this can be difficult for mothers and about 1 of 8 women living with HTLV-1 report breastfeeding for longer than recommended [32,33]. It is also important to consider other factors, in addition to the duration of breastfeeding, that may modulate the risk of transmission. Of note, considering short-term breastfeeding (up to 3 months) would not impact the cost-effectiveness results. 

As the proposed strategy will be targeting mainly ethnic minority and vulnerable groups, additional support is welcomed. In the UK, government support is available to low-income families as part of the Healthy Start scheme, which provides benefits and tax credits [34]. However, provision of free infant formula may be needed, and could be an expansion of HIV policies, as seen in other countries such as Brazil. The cost to provide free infant formula was included in our model [35]. These are challenges that need to be overcome to implement effective policies and should not be arguments not to implement the proposed policy.

One limitation of our study is the prevalence data used, which was extracted from the single study available that evaluated the prevalence in UK pregnant women in 2000 [12]. In the absence of public health policies, such as awareness and screening, and with the increase in immigration of people from HTLV-1 high endemic areas to the UK, a decrease in HTLV-1 prevalence in pregnant women would not be expected. Indeed, data presented recently at the HTLV-1 European Research Network meeting [30], indicate that the number of HTLV-1 infections diagnosed in the UK is increasing. In addition, we have used a range for prevalence in our sensitivity analysis and nearly all simulations were effective and cost-saving. Indeed, nearly all (99.9%) of the 1,000 simulations carried out in the probabilistic sensitivity analysis confirmed that screening is a dominant strategy, meaning it is more effective and less costly than not screening.

## Conclusion

HTLV-1 targeted antenatal screening could prevent 19 to 164 infant infections every year in England and Wales. Our analysis showed that screening is more effective and less costly than not screening, and therefore it should be considered for implementation. Systematic testing of pregnant women who have high risk of being infected by HTLV-1 would reduce HTLV-1 transmission in those ethnic minority groups and will contribute to improve health equity in the UK and potentially in other European countries with a similar epidemiological profile.

## Supplementary Data

295000 Supplement
