# Supplementary material for: Impact and economic analysis of human T-cell lymphotropic virus type 1 (HTLV-1)-targeted antenatal screening, England and Wales, 2021
Source: Euro Surveill. 2024 May 30;29(22):2300537. doi: 10.2807/1560-7917.ES.2024.29.22.2300537 (PMC11141129; doi:10.2807/1560-7917.ES.2024.29.22.2300537)
Supplement: Supplement [file 23-00537_ROSADAS_Supplement.pdf]

Impact and economic analysis of human T-cell lymphotropic virus type 1 (HTLV-1)-targeted antenatal screening, England and Wales, 2021

Carolina Rosadas*, Milene Costa, Kátia Senna, Marisa Santos, Graham P Taylor

Disclaimer: This supplementary material is hosted by Eurosurveillance as supporting information alongside the article Impact and economic analysis of HTLV-1 targeted antenatal screening in England and Wales", on behalf of the authors, who remain responsible for the accuracy and appropriateness of the content. The same standards for ethics, copyright, attributions and permissions as for the article apply. Supplements are not edited by Eurosurveillance and the journal is not responsible for the maintenance of any links or email addresses provided therein.

Supplementary Table S1. Prevalence of infections and diseases or syndromes that are screened for in the antenatal and neonatal care in the United Kingdom, in 2024 compared with HTLV-1 infection.

Infection or disease/syndrome	Positive tests	Number tested	Prevalence per 10,000	95% CI
HTLV-1 (parents born in the Caribbean)	NA		169.0	92–283
Hepatitis B	2,214	642,448	34.5	33–36
HTLV-1 (parents born in other endemic area)	NA		32.0	15–59
Down's syndrome	NA		26.5	25–28
Syphilis	1,019	642,433	15.9	15–17
HIV	621	642,434	9.7	9–10
Congenital hypothyroidism	443	591,851	7.5	7–8
Edward's' syndrome	NA		7.4	7–8
Sickle cell disease (newborns tested)	227	589,542	3.9	NA
HTLV-1 (overall prevalence in the UK)	NA		3.1	2–54
Cystic fibrosis	165	593,581	2.8	2–3
Patau's syndrome	NA		2.7	2–3
Phenylketonuria (PKU)	69	591,947	1.2	0–1
Medium-chain acyl-CoA dehydrogenase deficiency (MCADD)	40	591,946	0.7	0–1
Isovaleric acidaemia (IVA)	21	591,946	0.4	NA
Maple syrup urine disease (MSUD)	3	591,946	0.1	0–1
Glutaric aciduria type 1 (GA1)	3	591,947	0.1	NA
Homocystinuria (HCU)	1	591,946	0.02	NA

CI: Confidence Interval, HTLV-1: Human T cell lymphotropic virus type 1, NA: Not available, UK: United Kingdom.

References:

Ades AE, Parker S, Walker J, Edginton M, Taylor GP, Weber JN. Human T cell leukaemia/lymphoma virus infection in pregnant women in the United Kingdom: population study. BMJ. 2000;320(7248):1497-501. http://dx.doi.org/10.1136/bmj.320.7248.1497 PMID:10834889

Office for Health Improvement and Disparities. NHS Fetal Anomaly Screening Programme (FASP). NHS screening programmes in England: 2020 to 2021. London: gov.uk. [Accessed: 17 May 2024]. Available from: https://www.gov.uk/government/publications/nhs-screening-programmes-annual-report/nhs-screening-programmes-in-england-2020-to-2021#nhs-fetal-anomaly-screening-programme-fasp</eref>

National Health Service (NHS). NCARDRS Congenital Anomaly Official Statistics Report, 2020. London: NHS England; 2022. Available from: https://digital.nhs.uk/data-and-information/publications/statistical/ncardrs-congenital-anomaly-statistics-annual-data/ncardrs-congenital-anomaly-statistics-report-2020/prevalence-t21-t18-t13#top</eref>

National Screening Committee. Antenatal screening programme: HTLV. London: gov.uk; 2022. Available from: https://view-health-screening-recommendations.service.gov.uk/htlv</eref>

Supplementary Table S2. Live births in England and Wales (2021) from parents born in HTLV-1 endemic areas.

Live births England and Wales, 2021	
**Country of birth of mothers (high prevalence of HTLV-1)**	
Western Africa	10,487
Central Africa	1,838
Southern Africa	3,018
Central America	494
South America	4,238
Caribbean	2,124
Middle East	8,676
India	15,260
Pakistan	15,791
Romania	15,894
**Total**	**77,820**
**Country of birth of fathers (partners of mothers from low prevalence area)**	
Western Africa	3712
Central Africa	600
Southern Africa	2149
Central America	191
South America	1180
Caribbean	1956
Middle East	3093
India	2010
Pakistan	6285
**Total**	**21,176**
**Total to be screened**	**98,996**
**Total number of pregnant women England and Wales (2021)**	**624,628**

Supplementary Table S3. Live births (numbers and percentages) for the ten most common countries of birth of mother for non-UK-born mothers, England and Wales in 2021

Ref: Parents' country of birth - Office for National Statistics (ons.gov.uk)

Country of birth of mother	**2021** Rank	**2021** Number
Romania	1	15,894
Pakistan	2	15,791
India	3	15,260
Poland	4	13,373
Bangladesh	5	6,790
Nigeria	6	5,907
Germany	7	3,511
Lithuania	8	3,499
United States of America	9	3,446
Albania	10	3,260

Supplementary Table S4. High HTLV prevalence countries according to ECDC report

Geographical distribution of areas with a high prevalence of HTLV-1 infection (2015)

America
Argentina
Barbados
Brazil
Chile
Colombia
Dominican Republic
French Guyana (France)
Guadaloupe (France)
Guyana
Haiti
Jamaica
Martinique (France
Panama
Peru
Suriname
Trinidad and Tobago
Venezuela

Europe
Romania

Africa
Benin
Burkina Faso
Cameroon
Central African Republic
Chad
Congo
Cote d'Ivoire
Democratic Republic of the Congo
Equatorial Guinea
Gabon
Gambia
Ghana
Guinea
Guinea-Bissau
Liberia
Mali
Mauritania
Mozambique
Nigeria
Senegal
Seychelles
Sierra Leone
South Africa
Togo

Arabian Peninsula and Asia
Iran
Japan
Taiwan

Supplementary Table S5. HTLV-1 prevalence in pregnant women in the UK according to the country of origin:

Reference: Ades et al 2000.

	Anti-HTLV antibody	
	Total tested	Prevalence (per 1000)
**Africa**		
Western	2325	3-3.4
Central	559	3.65
Rest of Africa	4250	0
**America**		
Caribbean	914	16.4-17.5
South	490	2
Central and North	801	0
**Asia**		
Japan	233	4.3
Rest of Asia	12590	0
**Europe**		
UK	69013	0.33-0.38
Other Northern	1948	0
Southern	1143	0
Eastern	494	0
Western	1204	0.8
Oceania	529	0

Supplementary Table S6. Life Table UK

Reference: Office for National Statistics accessed 09 May 2023

*Average qX (i.e the mortality rate between age x and (x +1), that is the probability that a person aged x exact will die before reaching age (x +1)) for male and female was calculated.

age	qx
0	0.0038635
1	0.0002215
2	0.0001205
3	0.0000985
4	0.000075
5	0.000074
6	0.000078
7	0.000061
8	0.0000635
9	0.0000555
10	0.000072
11	0.000066
12	0.0000795
13	0.0001015
14	0.0001125
15	0.0001425
16	0.000168
17	0.0002345
18	0.00031
19	0.000333
20	0.000356
21	0.000359
22	0.000371
23	0.0003695
24	0.0003895
25	0.0004305
26	0.000432
27	0.0004695
28	0.0004975
29	0.000533
30	0.000579
31	0.0006145
32	0.000663

age	qx
33	0.000725
34	0.000787
35	0.000846
36	0.0009165
37	0.001056
38	0.0010545
39	0.0011625
40	0.001265
41	0.001352
42	0.001475
43	0.001627
44	0.0017775
45	0.0019755
46	0.0021355
47	0.0022435
48	0.002463
49	0.002674
50	0.0029055
51	0.0031365
52	0.003328
53	0.003583
54	0.003842
55	0.0041645
56	0.004615
57	0.004994
58	0.005531
59	0.005973
60	0.0065795
61	0.007136
62	0.008026
63	0.008685
64	0.009275
65	0.0102865
66	0.0113115

age	qx
67	0.012168
68	0.013475
69	0.0147035
70	0.0160665
71	0.017216
72	0.0191405
73	0.021544
74	0.024232
75	0.027033
76	0.0300635
77	0.034027
78	0.038304
79	0.042986
80	0.047877
81	0.0535785
82	0.059697
83	0.0673205
84	0.076219
85	0.085612
86	0.0973705
87	0.109363
88	0.122726
89	0.138061
90	0.1499775
91	0.1683815
92	0.1859475
93	0.2062745
94	0.226238
95	0.249589
96	0.2718325
97	0.2959945
98	0.317578
99	0.344926
100	0.370624

Detailed information of inputs used in the model

Incidence of breastfeeding in the UK

Asian (95%), Black (96%), or Chinese or other ethnic origin (97%) ,mixed race (89%), White (79%).

Breastfeeding rates UK

https://sp.ukdataservice.ac.uk/doc/7281/mrdoc/pdf/7281_ifs-uk-2010_report.pdf

Cost of screening test: £1.4 per test

*Based on quote for Murex anti-HTLV-1/2 ELISA £670 for 480 tests.

Cost of Western blot: £ 19.75 each

* Based on quote for HTLV Blot 2.4 £711 for 36 tests

Cost of formula up to 6 months

The cost of 800g formula powder is £9.9 (Sainsbury 10/05/2023) and £10.5 (Tesco 10/05/2023). In Brazil the recommendation is 66-77 formula for a baby up to 6 months (Portaria RN).

This results in a total cost of respectively £653.4 - £762.3 or £693 - £808.5

https://static1.squarespace.com/static/5efa4a95af311446a53c8cab/t/5fd0990c5347e801a823f769/1607

Cost of formula up to 12 months

Initial 66-77 formula up to 6 months followed by 72 (12 units x 6 months, introduction solid foods) = 138-149 units.

Total cost is £1,366.2 - £1,475.1 or £1,449 - £1,564.5

Cost of formula 3-6months

36-42 units of Formula £356.4 - 415.8 and £378 - 441

Cost of formula 3-12months

36-42 +72 equates to 108-114 £1069.2- 1128.6 or £ 1134 - 1197

Cost of Cabergoline

1mg tablets: 20 units costs 6425 p

1 tablet = 321.25 = 3.22 pounds

Ref: NHS Electronic Drug Tariff accessed in 12th May 2023

Breastfeeding incidence:

96% Black Caribbean NHS Digital. Infant Feeding Survey – UK, 2010.

Breastfeeding prevalence at 6 months:

61% Black Caribbean NHS Digital. Infant Feeding Survey – UK, 2010.

Utility values in the UK

Asymptomatic infection (Mean (SD)): 0.7802 (0.209) Rosadas et al 2020

HAM (Mean (SD)): 0.2025 (0.297) Rosadas et al 2020

Utility values people in the UK: 0.91 Janssen et al 2018

Annual Cost of Treating HTLV-1 Asymptomatic patient

£2900 (Imperial College Healthcare NHS Trust Resource Group)

Annual Cost of Treating HTLV-1 Associated Myelopathy (HAM)

£16000 (Imperial College Healthcare NHS Trust Resource Group)

Annual Cost of Treating Adult T cell Leukaemia

*Annual Cost of Treating Aggressive ATLL

£18016 (Imperial College Healthcare NHS Trust Resource Group)

*Annual Cost of Treating Indolent ATLL

£12907 (Imperial College Healthcare NHS Trust Resource Group)

75% Aggressive + 25% Indolent = 13512+3226.75 = £16,739
